# Supplementary material for: Psychrophrynella glauca sp. n., a new species of terrestrial-breeding frogs (Amphibia, Anura, Strabomantidae) from the montane forests of the Amazonian Andes of Puno, Peru
Source: PeerJ. 2018 Feb 27;6:e4444. doi: 10.7717/peerj.4444 (PMC5833480; doi:10.7717/peerj.4444)
Supplement: Supplemental Information 1 [file peerj-06-4444-s001.docx]

*Noblella madreselva* (2 specimens): PERU: Cusco: Provincia La Convención, Madre Selva (Santa Ana), CORBIDI 15769–70.

*Noblella pygmaea* (15 specimens): PERU: Cusco: Provincia Paucartambo, Kosñipata, MHNG 2725.29–30, MUSM 24535–36, 26306–7, 26318–20, 30423–24, 30453–54, MTD 47286–87.

*Psychrophrynella bagrecito* (14 specimens): PERU: Cusco: Quispicanchis: Marcapata, Río Marcapata, below Marcapata, ca. 2740 m, KU 196512 (holotype), KU 196513–18, 196520–21, 196523–25 (all paratypes); La Convención: Hacienda Huyro between Huayopata and Quillabamba, 1830 m, KU 196527–28.

*Psychrophrynella chirihampatu* (27 specimens): PERU: Cusco: Provincia Paucartambo, Área de Conservación Privada (ACP) Ukumari Llaqta, Comunidad Campesina de Japu, 2730 – 3000 m, CORBIDI 16495–16499, CORBIDI 16501–16509, CORBIDI 16696, MHNC 14656, MHNC 14658, MHNC 14661–14662, MHNC 14664, MHNC 14666–14672.

*Psychrophrynella usurpator* (78 specimens): PERU: Cusco: Provincia Paucartambo, Kosñipata, MUSM 20011, 20873–81, 20896–20913, 20925–33, 20946–47, 20955–57, 21012–18, 26272–73, 26278–79, 26308, 27592, 27906, 27950, 28033–28047, 30303, 30305, 30396–30400, 30405–30409, 30471–30474.
