# Supplementary material for: Psychrophrynella glauca sp. n., a new species of terrestrial-breeding frogs (Amphibia, Anura, Strabomantidae) from the montane forests of the Amazonian Andes of Puno, Peru
Source: PeerJ. 2018 Feb 27;6:e4444. doi: 10.7717/peerj.4444 (PMC5833480; doi:10.7717/peerj.4444)
Supplement: Supplemental Information 2 — Genbank accession numbers for the taxa and genes sampled in this study. [file peerj-06-4444-s002.docx]

| **Taxon** | **Voucher Nbr.** | **16S** |
| --- | --- | --- |
| *Barycholos pulcher* | KU 217781 | EU186709 |
| *Bryophryne bakersfield* | MHNC 5999 | KT276289 |
| *Bryophryne bustamantei* | MHNC 6019 | KT276293 |
| *Bryophryne cophites* | KU 173497 | F493537 |
| *Bryophryne phuyuhampatu* | CORBIDI 18226 | MF419256 |
| *Bryophryne quellokunka* | MUBI 5375 | MF186389 |
| *Bryophryne tocra* | MUBI 5419 | MF186398 |
| *Holoaden luederwaldti* | MZUSP 131872 | EU186710 |
| *Microkayla boettgeri* | MNCN43776 | MF186351 |
| *Microkayla chaupi* | MNCN43763 | MF186418 |
| *Microkayla chilina* | MNCN43772 | MF186414 |
| *Microkayla guillei* | AMNH-A 165108 | AY843720 |
| *Microkayla wettsteini* | KU 183049 | EU186696 |
| *Noblella heyeri* | QCAZ 31471 | JX267541 |
| *Noblella lochites* | KU 177356 | EU186699 |
| *Noblella myrmecoides* | QCAZ 40180 | JX267542 |
| *Noblella pygmaea* | MUSM 24536 | KY652645 |
| *Noblella* sp. | N/A | AM039646 |
| *Psychrophrynella chirihampatu* | CORBIDI 16495 | KU884559 |
| *Psychrophrynella glauca* | MUBI 16322 | MG837567 |
| *Psychrophrynella glauca* (holotype) | CORBIDI 18729 | MG837565 |
| *Psychrophrynella glauca* | MUBI 16323 | MG837568 |
| *Psychrophrynella glauca* | CORBIDI 18730 | MG837566 |
| *Pyschrophrynella usurpator* | KU 173495 | F493714 |
| *Strabomantis sulcatus* | KU 218055 | EF493536 |
